# Communication is key: extracellular vesicles as mediators of infection and defence during host–microbe interactions in animals and plants

**DOI:** 10.1093/femsre/fuab044

**Published:** 2021-08-27

**Authors:** Henrik U. Stotz, Dominik Brotherton, Jameel Inal

**Affiliations:** School of Life and Medical Sciences, University of Hertfordshire, Hatfield AL10 9AB, UK; School of Life and Medical Sciences, University of Hertfordshire, Hatfield AL10 9AB, UK; School of Life and Medical Sciences, University of Hertfordshire, Hatfield AL10 9AB, UK; School of Human Sciences, London Metropolitan University, London N7 8DB, UK

**Keywords:** biotroph, cellular cross-kingdom communication, endophytic, ESCRT, oomycete, pathogenesis

## Abstract

Extracellular vesicles (EVs) are now understood to be ubiquitous mediators of cellular communication. In this review, we suggest that EVs have evolved into a highly regulated system of communication with complex functions including export of wastes, toxins and nutrients, targeted delivery of immune effectors and vectors of RNA silencing. Eukaryotic EVs come in different shapes and sizes and have been classified according to their biogenesis and size distributions. Small EVs (or exosomes) are released through fusion of endosome-derived multivesicular bodies with the plasma membrane. Medium EVs (or microvesicles) bud off the plasma membrane as a form of exocytosis. Finally, large EVs (or apoptotic bodies) are produced as a result of the apoptotic process. This review considers EV secretion and uptake in four eukaryotic kingdoms, three of which produce cell walls. The impacts cell walls have on EVs in plants and fungi are discussed, as are roles of fungal EVs in virulence. Contributions of plant EVs to development and innate immunity are presented. Compelling cases are sporophytic self-incompatibility and cellular invasion by haustorium-forming filamentous pathogens. The involvement of EVs in all of these eukaryotic processes is reconciled considering their evolutionary history.

## Teaser

Within the context of animal extracellular vesicles (EVs) and their interactions, recent developments in the growing fields of plant and fungal EVs suggest complex cross-kingdom communication with microbes for infection/protection.

## INTRODUCTION

The endomembrane system was discovered in the mid-1940s, aided by newly developed cell fractionation and electron microscopy (EM) techniques, leading the way to understand the traditional secretory pathway with the help of pancreatic exocrine cells (Palade 1975). In the meantime, insights were made into intercellular communication as well as with the extracellular environment, which is crucial in many cellular processes including cell survival, differentiation, proliferation and apoptosis. An understanding of this form of communication has led to the establishment of the role of extracellular vesicles (EVs) as mediators of such communication by facilitating the exchange of growth factors, enzymes, cytokines and various other signalling molecules. As far back as 1946, EVs were first reported as a precipitable factor. In the later coagulation research of Peter Wolf, this was called 'platelet dust' (Wolf 1967), able to accelerate the secretion of thrombin in platelet-free plasma and was subsequently observed by EM to bud from activated platelets. The receipt of these many signals is then part mediated by the endosomal system consisting of three networks of membranes within cells: (i) primary endocytic vesicles, (ii) early endosomes and (iii) multivesicular bodies (MVBs). Endocytosed molecules, including surface proteins upregulated during activation, taken up via primary endocytic vesicles and early endosomes, are then recycled directly to the plasma membrane or trafficked to MVBs. Receptors and other transmembrane proteins are targeted to the intraluminal vesicles (ILVs) of MVBs for degradation, thus allowing for removal of excess proteins, by fusion of the MVBs with lysosomes. However, as will be reviewed in this article, some proteins, rather than following a degradative pathway via lysosomes through their association with ILVs, can upon MVB fusion with the plasma membrane be released into the extracellular space as small EVs (sEVs, formerly termed exosomes). The contents of sEVs and medium EVs (mEVs, formerly microvesicles) that bud off from the plasma membrane comprise a range of active biomolecules including nucleic acids (e.g. small and long noncoding RNAs and mRNA), proteins and lipids (Inal *et al*. 2013b; Leidal *et al*. 2020). Fungal EVs also carry tRNA (Peres da Silva *et al*. 2015b). Constitutively released membrane vesicles (MVs) from Gram-negative and certain Gram-positive bacteria carry peptidoglycans, phospholipids, lipopolysaccharides, outer membrane proteins, various soluble (periplasmic and cytoplasmic) proteins and nucleic acids. This content can vary according to growth conditions (Dauros Singorenko *et al*. 2017).

Secretion of EVs by fungi and plants was noted in the 1960s. Hyphae of true fungi (Eumycota) were shown to secrete vesicles, termed lomasomes, that looked and behaved a lot like MVBs (Moore and McAlear 1961). MVBs were later shown and correctly identified in meristematic cells of carrot (*Daucus carota*) cell suspension cultures (Halperin and Jensen 1967). Similar to the earlier study in fungi, MVBs were noted to fuse with the plasma membrane, releasing their contents into the cell wall. This review will discuss the progress that has been made since these pioneering studies to better understand EV biogenesis and function in plants and fungi and their relationship to cross-kingdom interactions.

### Vesicles as thermodynamic entities

All living cells vesiculate, allowing for intracellular and extracellular compartmentalization and the evolutionary fitness this entails. However, the integral role of vesiculation in cellular life has emerged gradually. Following the formalization and universal adoption of cell theory throughout the 18th and 19th centuries, the initial conception of a dynamic and polymorphous cell membrane dates to suggestions made by late 19th century physician Quincke, who posited that fluid fats must be their chief constituent, based on observations that during plasmolysis of plant cells, the protoplasm 'frequently breaks up into two or more balls, which spread themselves out, and then either re-unite, or remain separated … just as two soap bubbles' (Hertwig 1895).

Through the sometimes surreptitious determination of membrane thickness (Fricke 1925), bilayer structure (Gorter and Grendel 1925) and barrier properties (Danielli and Davson 1935), competing theories eventually culminated in the development of modern cell membrane theory, along with the observation that amphipathic phospholipids spontaneously self-assemble into unilamellar micelles and bilayered vesicles in aqueous solution (Hill 1964; Hall and Pethica 1967; Tanford 1973). By the 1970s, the underlying thermodynamics, hydrophobic and intermolecular forces, free-energy considerations and molecular geometry of this process were broadly understood to account for spontaneous self-assembly, as well as vesicle size distribution and bilayer elasticity (Israelachvili, Mitchell and Ninham 1977).

Vesicle thermodynamics continue to be a contemporary topic of interest with both *in vitro* experimentation and *in silico* computer modelling showing not only that spontaneous vesiculation from phospholipid membranes is correlated with membrane thickness but also that vesicle fission and fusion may be energetically permitted without the need for regulation or protein machinery (Dobereiner *et al*. 1993; Markvoort and Marrink 2011; Huang *et al*. 2017). Additionally, transmission EM (TEM) and nuclear magnetic resonance data have elucidated novel self-assembling lipid-protein and lipid-DNA topologies such as hexagonal (Allain, Bourgaux and Couvreur 2012) and various cubic conformations (Conn and Drummond 2013).

Indeed, current evolutionary theories extend this theoretical trajectory to describe self-assembled vesicles as an entropic ‘stepping stone' from abiotic, geochemical substrates to complex biochemistry and primitive cells (Chen and Walde 2010), highlighting the role of vesiculation in the evolution of protocells, the last universal common ancestor (LUCA), and enveloped viruses (Szathmáry, Santos and Fernando 2005; Budin, Bruckner and Szostak 2009; Errington 2013; Nolte-’t Hoen *et al*. 2016).

### Intra- and extracellular vesicles

Despite much fundamental research, the roles of vesicles in cellular communication remained obscure until the late 20th century, with most work focusing on intracellular vesicle communication. Through the Nobel prize-winning work of Randy Schekman, James Rothman and Thomas Südhof, it was discovered that intracellular vesicles of eukaryotes comprise a fundamental part of the endomembrane system, trafficking cargo between the nuclear envelope, endoplasmic reticulum (ER), Golgi and plasmalemma (Kaiser and Schekman 1990; Hata, Slaughter and Sudhof 1993; Sollner *et al*. 1993). As such, specialized vesicles, such as lysosomes, endosomes and autophagosomes, are often categorized as separate organelles within this system (Harris 1986). Many of these complex sorting pathways are now broadly described, at least in model organisms (Nebenfuhr 2002; Hu *et al*. 2015; Palmisano and Melendez 2019).

Comparably, the EVs of eukaryotes have not until recently enjoyed the same limelight, while carrying no less complexity in terms of trafficking pathways. Indeed, it is tempting to speculate that when considering the ability for EVs to engage in cross-kingdom communication, it may ultimately be found that EVs represent a greater diversity of messages than their evolutionarily conserved intracellular counterparts. Despite initial neglect, EVs of animals and all other kingdoms are now relatively well studied. Discussing all varieties of protist and prokaryotic EVs is beyond the scope of this review, with each deserving its own dedicated space. Instead, the focus of this review shall be to compare and contrast the three multicellular eukaryotic kingdoms of animals, plants and fungi and explore their interactions.

## EXTRACELLULAR VESICLES

### EVs in humans and animals as a paradigm

Since the 1940s it has been known that human plasma contains a subcellular component facilitating fibrin formation (Chargaff and West 1946; O'Brien 1955). Later, through the use of EM, it was possible to show that these subcellular factors comprised microscopic vesicles, originally termed ‘platelet dust’, nowadays EVs, and that they possessed procoagulant activity, similar to that provided by intact platelets (Wolf 1967).

More recently and since the formation of the International Society for Extracellular Vesicles (Araldi *et al*. 2012) the interest in EVs has grown exponentially (Srivastava *et al*. 2020). Progressing from an initial interest in their procoagulant properties, they were found to play roles in inflammation (Freyssinet *et al*. 1999; Nieuwland and Sturk 2002), and the circulating EVs in blood were found to be derived from a range of cells including platelets, erythrocytes, lymphocytes, granulocytes, monocytes and endothelial cells. Many pathogens also release EVs as a decoy function to prevent the deposition of complement or to activate and consume complement in the surroundings as was found with the unicellular protozoan parasite, *Trypanosoma cruzi* (Cestari *et al*. 2012). Furthermore, the infection process, certainly for intracellular pathogens, stimulates release of EVs from host cells. As well as playing evasive strategies for example as decoys (Inal *et al*. 2013b), pathogens may opportunistically utilize host EVs to acquire complement inhibitors (Cestari *et al*. 2012; Inal, Ansa-Addo and Lange 2013a). The decoy function of EVs is not unique to animal cells as bacteria produce MVs for interception of bacteriophages (Toyofuku, Nomura and Eberl 2019). These bacterial MVs also carry enzymes that can degrade antibiotics (Schwechheimer and Kuehn 2015). Furthermore, just as outer membrane vesicles (OMVs) from *Porphyromonas gingivalis* may assist with the interaction of other periodontal bacterial pathogens with eukaryotic host cells (Kamaguchi *et al*. 2003), we found this to also be so with the intestinal parasite *Giardia intestinalis* whose EVs aided attachment to intestinal epithelial cells (Evans-Osses *et al*. 2017). EVs from protozoan parasites, such as *T. cruzi* shuttle genetic information between parasites and host cells. Fungal EVs meanwhile are rich in enzymes able to degrade the cell wall that likely explains their route across the cell wall, a similar problem to that faced by MVs from Gram-positive bacteria as well as several virulence factors as described later.

### Properties and mechanism of release of mEVs (microvesicles) and lEVs (apoptotic bodies)

**Figure 1.**
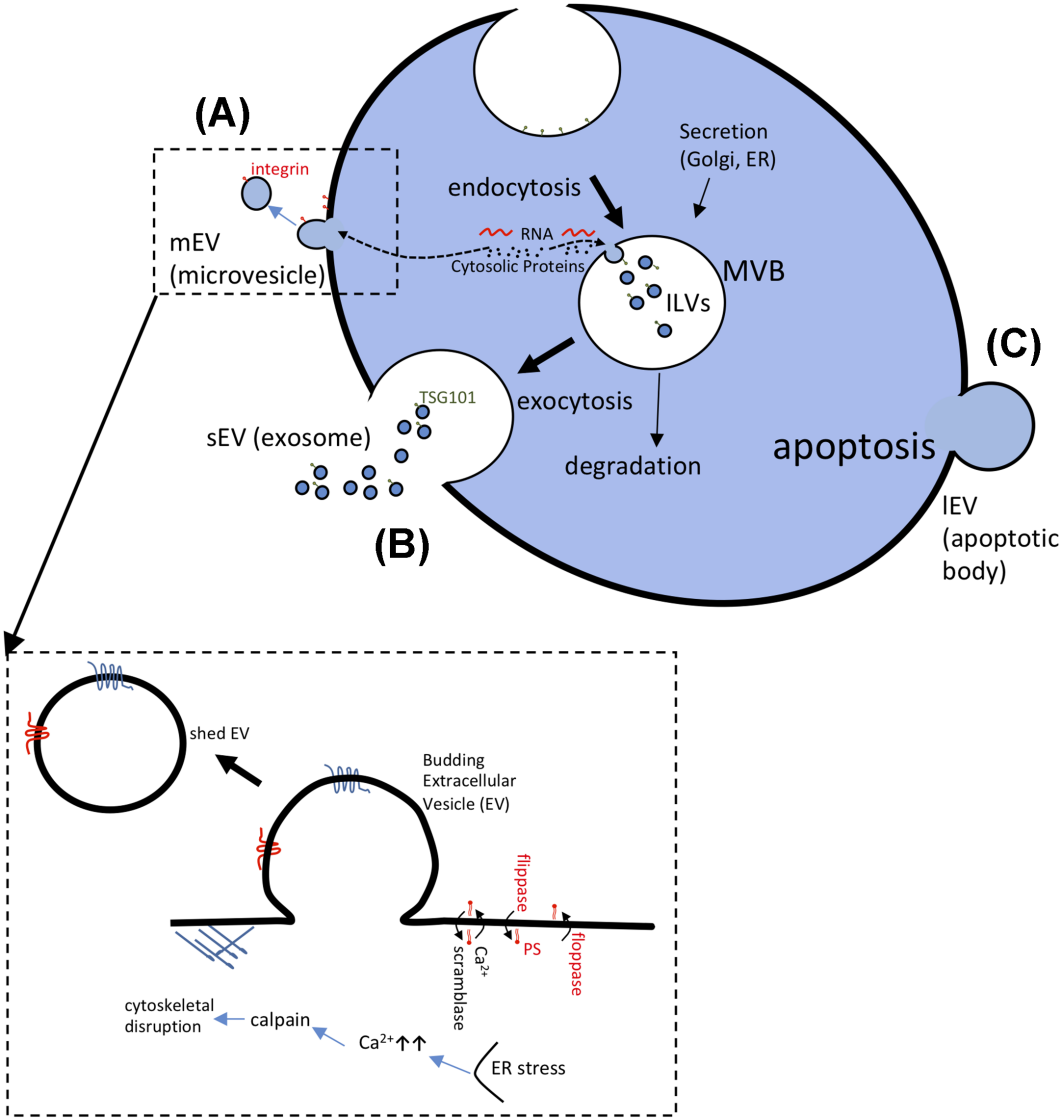
Biogenesis of microvesicles (mEVs), ILVs, exosomes (sEVs) and apoptotic bodies (lEVs) in animals. **(A)** mEVs are shed from the plasma membrane and shown in larger scale as a result of increased [Ca^2+^]_i_, cytoskeletal disruption and loss of lipid asymmetry. **(B)** sEVs are formed by intraluminal budding of late endosomes/MVBs and released upon their fusion with the plasma membrane. TSG101 is a protein involved in ILV biogenesis. **(C)** lEVs (apoptotic bodies) are released from the cell surface during apoptosis. Although evidence suggests mEV biogenesis, sEVs are more commonly generated in fungi and plants. Cellular structures are not drawn to scale.

According to MISEV2018 (Thery *et al*. 2018) EVs comprise the small sEVs and medium mEVs as well as large EVs (lEVs or apoptotic cell-derived EVs). mEVs are phospholipid-rich, microscopic vesicles formed by exocytic budding of the plasma membrane (Fig. 1). During EV formation, the lipid asymmetry of the lipid bilayer, which comprises phosphatidylserine (PS), phosphatidylethanolamine (PE), phosphatidylcholine (PC) and sphingomyelin (SM) is lost, resulting in an outer leaflet that is rich in negatively charged phospholipids. Whilst the neutral phospholipid PC and SM are primarily located on the outer leaflet of the lipid bilayer, the negatively charged PS and PE are located to the inner leaflet. This asymmetrical distribution of phospholipids in the plasma membrane is actively maintained by various enzymes, including aminophospholipid translocase (APT, flippase) or floppase (Sims and Wiedmer 2001), but also scramblase, calpain and gelsolin (the latter present only in platelets) (Piccin, Murphy and Smith 2007). The lipid asymmetry is maintained by these enzymes allowing membrane phospholipids to move to the outer leaflet whilst the aminophospholipids are simultaneously redirected to the inner leaflet of the bilayer (Piccin, Murphy and Smith 2007). When cells become activated or during early apoptosis the ability to maintain this asymmetric distribution of the lipid bilayer is lost. Negatively charged phospholipids such as PS and PE are then exposed at the membrane surface. When intracellular concentrations of calcium rise for example during activation of cells (Stratton *et al*. 2015), infection by intracellular pathogens, or sublytic deposition of calcium ionophore or of complement proteins as a membrane attack complex, then the steady state is changed resulting in PS expression on the membrane surface (Fox *et al*. 1990; Connor *et al*. 1992; Diaz and Schroit 1996).

The intracellular mechanism(s) leading to mEV biogenesis are not fully elucidated, but the process does seem to be dependent on an underlying stimulus. There may even be multiple biogenesis pathways depending on the stimulus, and mEV release may occur through either activation of cell death, whether apoptotic or necrotic (Ardoin and Pisetsky 2008). The signals that induce cell activation/apoptosis, include chemical stimuli, such as cytokines, endotoxin and thrombin, or physical stimuli, such as hypoxia or shear stress (Vanwijk *et al*. 2002), the latter typically being important in mEV release from platelets (Gasser *et al*. 2003). Other triggers would include complement membrane attack complex C5b-9, with or without antibodies, phorbol esters, calcium ionophore (A23187), adenosine diphosphate, adrenaline and microbial peptides such as formyl-methionyl-leucylphenylalanine (Gasser *et al*. 2003).

Cellular activation of platelets leads to mEV formation (Fig. 1) through a rise in cytosolic calcium and the concomitant activation of calpain and protein kinases, which causes cytoskeletal rearrangement, membrane blebbing and mEV formation (Wiedmer and Sims 1991; Yano *et al*. 1994; Miyazaki *et al*. 1996). mEVs may also be released *in vitro* by depriving cells of growth factor or through complement activation (Hamilton *et al*. 1990; Jimenez *et al*. 2003).

In apoptosis, lEV (or apoptotic body) release is associated with membrane blebbing, which involves a redistribution of cellular contents, likely due to changes in volume-induced stress during cell death perhaps related to volume stress that occurs as cells die. ROCK-1 (Rho associated kinase 1), an effector of Rho GTPases, is essential for apoptotic membrane blebbing, although not all cells bleb, and is activated during mEV biogenesis (Distler *et al*. 2005); indeed blebbing itself can differ during the different stages of apoptosis. In the terminal phases of apoptosis mEV release seems most likely to occur and this is likely to coincide with cell fragmentation and apoptotic body formation, which represents collapsed cells undergoing nuclear fragmentation. Differences in the mechanism of mEV formation are likely to depend on whether the cells are undergoing cell activation or apoptosis and such differences may consequently lead to variations in mEV size and macromolecular cargo (protein and RNA), which may also lead to functional differences.

### sEVs are generated through exocytosis

As for mEVs, sEVs play roles in maintaining normal cellular physiology as well as in disease pathology (Vlassov *et al*. 2012). In terms of biogenesis, sEVs have an endocytic origin. During endocytosis an early endosome is formed. This may then either follow a degradative pathway, upon fusion with lysosomes, or undergo intraluminal budding to generate ILVs within an MVB. Upon fusion of the MVB with the plasma membrane, its cargo of ILVs is released as sEVs (Fig. 1). There are two separate pathways that result in the formation of ILVs. For the inward budding process and cleavage of bud necks of the MVB limiting membranes, components of endosomal sorting complex required for transport (ESCRT) are involved (van Dommelen *et al*. 2012). In the second pathway known as the ESCRT-independent exosomal pathway, SMase results in the hydrolysis of sphingomyelin. This generates the cone shaped ceramide, which is believed to result in an immediate negative curvature on the cytosolic leaflet of the endosomal membrane. In turn this induces the inward budding into the endosome and formation of the ILVs (Hurley *et al*. 2010).

sEVs have a density in sucrose from 1.13 to 1.19 g cm^–3^ and as for mEVs share marker proteins with their parental cell (Inal *et al*. 2013b; Raposo and Stoorvogel 2013). Amongst characteristic marker proteins, distinctive for sEVs, and present in high abundance (Conde-Vancells *et al*. 2008; Subra *et al*. 2010), are heat shock proteins (Hsp90 and Hsc70), fusion proteins and membrane transport proteins (GTPases, annexins and flotillin), proteins involved in ILV biogenesis (TSG101 and Alix) and a range of tetraspanins (CD9, CD63, CD81 and CD82).

Given the generation of EVs during exocytosis (sEVs) or blebbing of membranes (mEVs), their origin can be tracked by cell-specific protein markers. The rules governing the incorporation of different proteins into EVs are not known. These EVs also carry antigens expressed on the surface of the mother cell (Lynch and Ludlam 2007). It is this anionic phospholipid surface that then mediates many of the biological functions of mEVs in animals including the binding of coagulation factors as well as the expression of functional molecules such as selectins or tissue factor.

### EV biogenesis in filamentous microbes

Understanding of EVs in other multicellular eukaryotes has lagged behind and it was not until this millennium that a general awareness of fungal and plant EVs has emerged (An, van Bel and Hückelhoven 2007; Rodrigues *et al*. 2007). Clear documentation of mEVs biogenesis in fungi is lacking. However, an EM study of protoplasts from *Aspergillus nidulans* first documented vesicles budding from the fungal plasma membrane (Gibson and Peberdy 1972). Further work on fungal protoplasts of *Aspergillus fumigatus* recently showed that specific EVs are generated via plasma membrane budding similar to mEV production in animals (Rizzo *et al*. 2020). The authors mentioned that the fungal cell wall might preclude the observation of vesicles budding from the plasma membrane reminiscent of mEVs biogenesis in fungi.

Conversely, definitive proof does exist for sEVs biogenesis from MVB in multicellular eukaryotes other than animals. The powdery mildew pathogen *Golovinomyces orontii* produces MVBs that fuse with the plasma membrane to release sEVs (Table 1) (Micali *et al*. 2011). The oomycete that caused the Irish potato famine, *Phytophthora infestans*, and the rice blast fungus *Magnaporthe oryzae* deliver effectors into the cytoplasm of their hosts via unconventional protein secretion pathways (Giraldo *et al*. 2013; Liu *et al*. 2014). Upon penetration of the rice epidermis, *M. oryzae* initially forms invasive hyphae (IH) that secrete apoplastic effectors via conventional secretion. IH also form biotrophic interfacial complexes (BICs) that accumulate cytoplasmic effectors via unconventional secretion.

**Table 1.** Evidence for involvement of extracellular vesicles in controlling biological processes.

Kingdom	Biological process/organism	Structure	Compatible reaction	Incompatible reaction, defence	References
Plant	Flower fertilization	Pollen grain	MVBs, EVs; *Exo70A1*^a^	Autophagy	Goring (2018)
Plant	Barley (interaction with *Bgh*^b^)	Haustorium	MVBs, 'mEVs', 'autophagy'^c^	HR, MVBs	An *et al*. (2006a)
Plant	Barley (interaction with *Bgh*^b^)	Haustorium		MVBs, 'Autophagy'^d^	An *et al*. (2006b)
Plant	Barley, *Arabidopsis* (penetration resistance)			sEVs; *PEN1*, *HvEXO70F*^e^	An, van Bel and Hückelhoven (2007); Ostertag *et al*. (2013)
Plant	*Arabidopsis* (*Botrytis* tolerance)	Penetration sites		sEVs, tetraspanin, sRNAs	Cai *et al*. (2018)
Plant	Barley (*Ramularia* interaction)		*ROR1*,*ROR2*^f^		McGrann *et al*. (2014)
Fungus	*Golovinomyces orontii*	Haustorium	MVBs, sEVs		Micali *et al*. (2011)
Fungus	*Blumeria graminis* f. sp. *hordei*	Appressorium, haustorium		MVBs	An *et al*. (2006a)
Fungus	*Magnaporthe grisea* (host penetration)	Appressorium	Tetraspanin ('sEVs')		Clergeot *et al*. (2001)
Fungus	*Magnaporthe oryzae* (host colonization)	BIC^g^	Autophagy, unconventional secretion		Sun *et al*. (2018)
Protist	*Histoplasma capsulatum*	Receptor-mediated endocytosis	Prevention of phagocytosis, apoptosis of host cell macrophage		Garfoot and Rappleye (2016)
Animal	*Homo sapiens*, natural killer cells and cytotoxic T-cells	Attack complex		EV release of perforins and granzymes	Schmidt, Tramsen and Lehrnbecher (2017); Di Pace *et al*. (2020); Del Vecchio *et al*. (2021)

a MVB, multivesicular body; EV, extracellular vesicle; molecular component involved in secretion is listed.

b *Bgh*,*Blumeria graminis* f. sp. *hordei*.

c In quotations: The authors suggest compartments/processes based on microscopic evidence; microvesicles (mEVs) may form at the extrahaustorial membrane, MVBs and vesicles were found in the central vacuole.

d In quotations: Published suggested cellular process.

e Examples of molecular components involved in penetration resistance employing exosomes (sEVs).

f Penetration resistance may have trade-offs regarding resistance against pathogens other than powdery mildew fungi.

g BIC, biotrophic interfacial complex.

### Molecular mechanisms of fungal EV formation

Secretory regulators and Snf7p, which are involved in MVB formation, influence the composition and release of EVs in yeast (Oliveira *et al*. 2010b; Russell *et al*. 2012). MVB formation is also dependent on the ESCRT complex. The ESCRT machinery determines the size, abundance and composition of EVs (Zhao *et al*. 2019). EV cargo enriched in cell wall remodelling enzymes protects against antifungal compounds (Zarnowski *et al*. 2018; Zhao *et al*. 2019). Vps20 and Snf7 are among the constituents of the ESCRT-III complex that cleaves off ILVs (Babst *et al*. 2002; Oliveira *et al*. 2013). Membrane curvature and budding of vesicles is dependent on APTs that contribute to lipid asymmetry (Farge *et al*. 1999). The P4-ATPase Drs2p is an APT involved in endo/exocytic pathways (Gall *et al*. 2002; Liu *et al*. 2008). Similarly, APT1 of *Cryptococcus neoformans* contributes to polysaccharide secretion via EVs and pathogenesis as well as the intracellular membrane architecture (Rizzo *et al*. 2014; Rizzo *et al*. 2018). A genetic screen in *Saccharomyces cerevisiae* resulted in identification of *snf7∆*, *vps20∆* and *drs2∆* as oxalate-sensitive mutants (Cheng *et al*. 2007). While these three genes are clearly involved in ILV and sEV formation, it remains to be determined whether they alter transport of oxalate to the vacuole, out of the cell or both. Besides, deletion of a putative phospholipid-translocating scramblase of *Cryptococcus gattii* increased the size and altered the composition of EVs; intracellular vesicles and membranes were affected as polysaccharide secretion and capsule formation were enhanced (Reis *et al*. 2019).

A loose consensus has developed that not only do fungi indeed release sEVs via an endosomal/exosomal, MVB-like mechanism, but also through at least one other independent process, analogous to mEV membrane budding (Oliveira *et al*. 2010b; Huang *et al*. 2012; Oliveira *et al*. 2013; Rodrigues *et al*. 2014; Bleackley *et al*. 2019). Experiments in *C. neoformans* showed that mutants lacking Golgi reassembly and stacking protein (GRASP) and the autophagy regulator Atg7 produce only sEVs, with authors suggesting these to be produced via unconventional secretion that bypasses autophagosomal and ESCRT/MVB pathways (Peres da Silva *et al*. 2018). Similar work in *S. cerevisiae* showed that while ESCRT proteins helped determine protein composition, they were not essential for EV release (Oliveira *et al*. 2010b).

### EV biogenesis in plants

**Figure 2.**
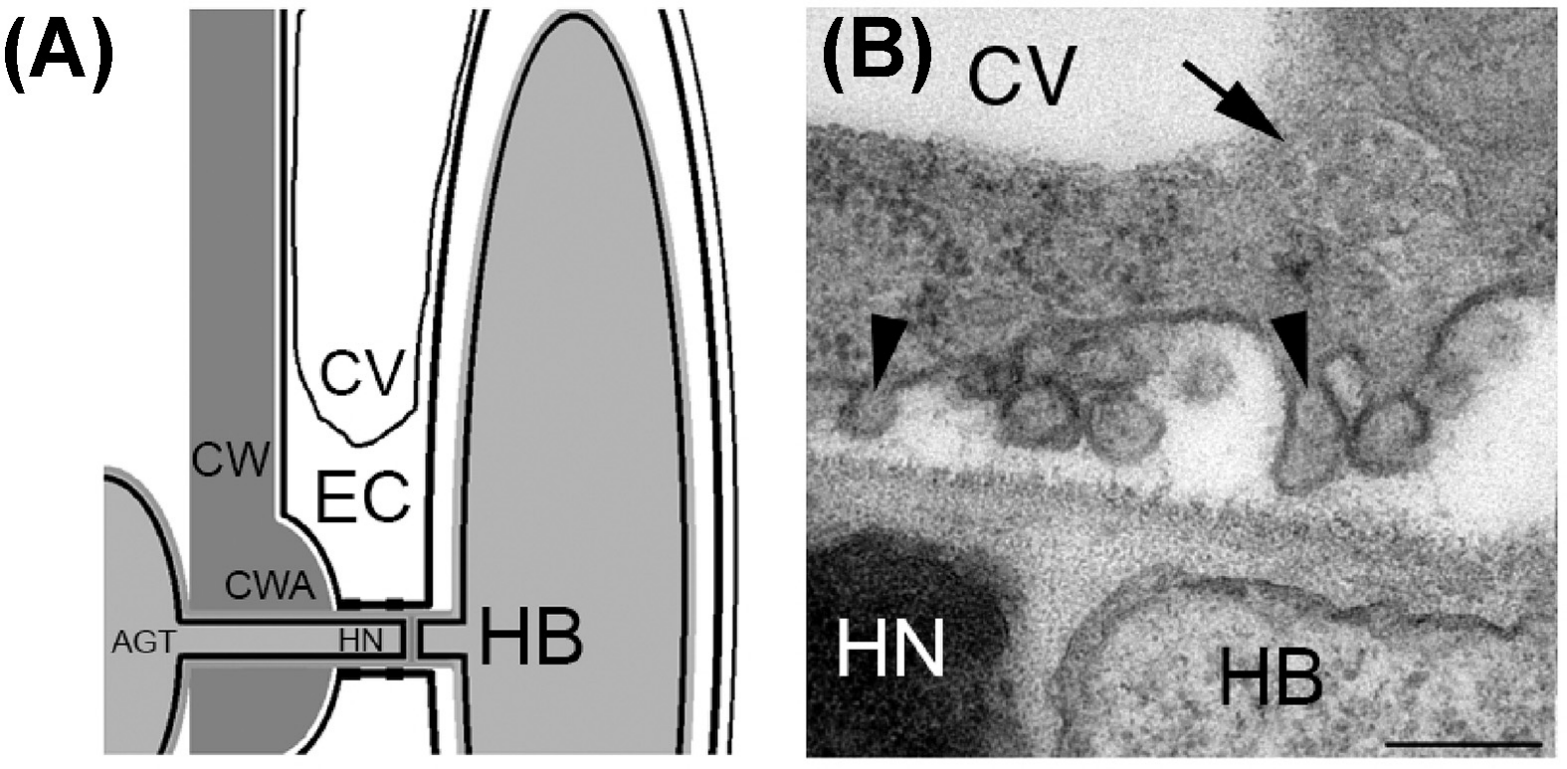
Microscopic evidence for mEV (microvesicle) formation in haustorium containing epidermal cells of powdery mildew (*Blumeria graminis* f. sp. *hordei*) infected susceptible barley cultivar Pallas (b) at 20–21 h postinoculation. **(A)** Schematic representation of cellular structures and compartments. **(B)** An MVB (arrow) near a haustorium. Arrowheads point to evaginations or protrusions of the extrahaustorial membrane; mEVs are formed by such evaginations or protrusions. AGT, appressorial germ tube; CV, central vacuole; CW, cell wall; CWA, cell wall apposition; EC, epidermal cell; HB, haustorial body; HN, haustorial neck; bar, 200 nm; from An *et al*. (2006b) with modifications.

Definitive information on mEV biogenesis in plants is lacking, although plant cells infected with fungus produce membrane evaginations (An *et al*. 2006b) into the extracellular matrix that is in contact with the invading pathogen, reminiscent of mEVs in animals (Fig. 2). In the absence of biomarkers, mEV biogenesis in plants remains speculative.

As in other eukaryotes, production of EVs in plants depends on the secretory pathway and involves the exocyst complex (Vukasinovic and Zarsky 2016; Picco *et al*. 2017) consisting of eight subunits (Safavian *et al*. 2015). Vesicle fusion is facilitated with the help of vesicle (v)-SNARE and target (t)-SNARE complexes. Vesicle secretion includes canonical and unconventional secretion pathways, the latter of which results in the release of sEVs and mEVs (Inal *et al*. 2013b). Extracellular fluids were collected from imbibed sunflower seeds to demonstrate the existence of vesicles with a diameter of 50–200 nm that contain a lectin and a Rab11 GTPase (Regente *et al*. 2009). Further analysis of imbibed sunflower seeds demonstrated unconventional secretion of the Helja lectin (Regente *et al*. 2012). EVs from sunflower seedlings were enriched in cell wall remodelling enzymes and defence proteins (Regente *et al*. 2017). Strikingly, PMR5 involved in pectin methyl esterification and susceptibility to penetration by powdery mildew pathogens (Vogel *et al*. 2004; Chiniquy *et al*. 2019) was associated with EVs (de la Canal and Pinedo 2018). Cell wall remodelling activities may allow EVs to pass through the cell wall and mediate or restrict other forms of transport or pathogen ingress. EVs from apoplastic fluids of *Arabidopsis thaliana* leaves were enriched in proteins involved in biotic and abiotic stress responses (Rutter and Innes 2017). Analysis of the xylem sap of tomato showed that the majority of proteins were not part of the canonical secretion pathway, suggesting the existence of unconventional secretion pathways (de Lamo *et al*. 2018). Sphingolipids were enriched in EVs from apoplastic fluids of *A. thaliana* leaves relative to whole leaf extracts (Liu *et al*. 2020). The majority of EV sphingolipids was composed of glycosyl inositol phosphoceramides and this negatively changed sphingolipid was less abundant in leaves of the *TETRASPANIN 8* (*tet8*) mutant relative to wild-type plants.

The predominant pathway of EV biogenesis in plants is via MVBs, and evidence for differences between tetraspanin (TET8)- and t-SNARE (PEN1)-positive putative sEVs were reported in *A. thaliana* (He *et al*. 2021); TET8- and PEN1-positive sEVs fractionate differently and differ in vesicular content. Additionally, exocyst-positive organelle (EXPO)-derived EVs were reported (Wang *et al*. 2010a); these putative sEVs were reportedly endosome-derived but not related to MVBs.

### Developmental control of vesicle secretion and sorting in plants

Plants constitutively secrete EVs (Regente *et al*. 2009; Rutter and Innes 2017) but also respond to biotic cues (Rutter and Innes 2017; Goring 2018). A well-documented developmental event controlled by stimulus-dependent vesicle trafficking is flower fertilization (Goring 2018). When self-incompatible pollen lands on a stigma, the *S*-locus protein 11/*S* cysteine-rich ligand is transferred from the pollen coat to the stigmatic papilla cell carrying the corresponding *S* receptor kinase (Watanabe, Suwabe and Suzuki 2012). The signal transduction pathway downstream of this molecular recognition event results in phosphorylation and activation of ARC1, an E3 ubiquitin ligase that targets the exocyst component Exo70A1 for degradation (Katashiba and Nasrallah 2014). Consequently, MVBs are targeted to the vacuole, accumulating in autophagic bodies (Table 1) (Safavian and Goring 2013; Goring 2018). Conversely, when compatible pollen lands on the stigmatic surface, small local calcium waves are initiated that precede pollen hydration, tube germination and penetration (Iwano *et al*. 2015). MVBs rapidly fuse with the plasma membrane to release sEVs into the stigmatic cell wall (Table 1) (Elleman and Dickinson 1996; Safavian and Goring 2013). As a result, the stigmatic cell wall in contact with the pollen grain expands in preparation for pollen penetration (Elleman and Dickinson 1996). As plant sEVs are enriched for aquaporins and cell wall degrading enzymes (Regente *et al*. 2017; Rutter and Innes 2017), their secretion probably contributes to pollen hydration, tube germination and penetration.

### Cellular uptake of EVs

**Figure 3.**
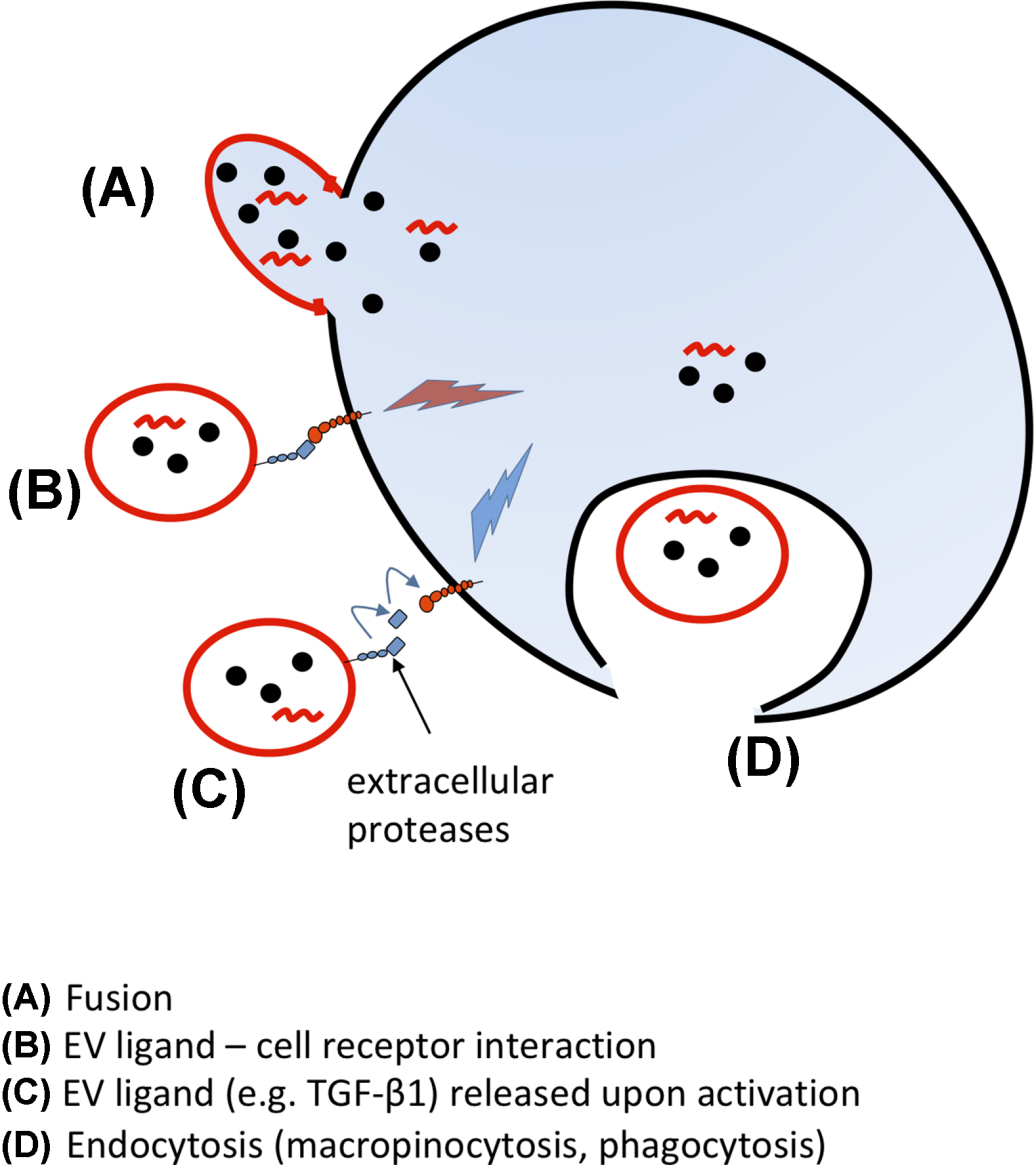
Interaction of EVs with target cells in animals. **(A)** EV interaction by membrane fusion released intravesicular contents into cytoplasm of target cell. Protein interaction between EV and target cell resulting in intracellular signalling of target cell (**B**) or activation of surface-bound proteins (**C**) or uptake (**D**) by endocytosis; structures are not drawn to scale. This figure highlights macromolecules; black dots and red waves refer to proteins and RNA, respectively. Although EV uptake is documented in fungi, the molecular basis is not understood. There is little, if any, evidence for EV uptake in plants.

There are four mechanisms by which EVs can interact with recipient cells (Fig. 3). These are (i) fusion, (ii) surface protein interaction, triggering signal transduction in the target cell, (iii) activation of an EV-bound surface protein and (iv) endocytosis.

Membrane fusion (Fig. 3A) is likely to be mediated by a prior interaction of surface proteins between EV and target cell. Adhesion proteins for example on endothelial progenitor cell-derived mEVs are thought to interact with fusion proteins on recipient endothelial cells to facilitate fusion (Hargett and Bauer 2013). Such fusion may also lead to the transfer of surface receptor proteins, resulting in particular cellular responses (Mause and Weber 2010; Meckes and Raab-Traub 2011). EVs and cells may also simply interact via receptor–ligand interactions (Fig. 3B), triggering signal transduction in the target cell but with no fusion or uptake. Another example of protein interactions without EV fusion or uptake (Fig. 3C) would be that following activation of an EV-bound transforming growth factor β (TGF-β), bound in a latent complex on the EV surface, by plasmin or integrin, releasing it to interact with its cognate receptor on the target cell. In terms of endocytosis, EVs may be taken up by ligand-mediated endocytosis or macropinocytosis (Costa Verdera *et al*. 2017).

### Uptake of EVs by fungal and plant cells

It was demonstrated that 60–80 nm liposomes could penetrate the fungal cell wall with a predicted pore size of <6 nm, suggesting that the cell wall is more dynamic than previously thought, with flexible viscoelastic properties permitting bi-directional vesicle traffic from and to cells (Walker *et al*. 2018). No mechanistic information on vesicle fusion/uptake in fungal cells is currently available, although TEM evidence suggests that the hydrophobic polyene antibiotic amphotericin B did promote liposome uptake and fusion in *Candida albicans*. The fungal pathogen *Sclerotinia sclerotiorum* rapidly internalizes EVs from the host plant sunflower (Regente *et al*. 2017). As a result, hyphal growth inhibition and abnormalities occurred as well as cell death. *Botrytis cinerea*, a pathogen related to *S. sclerotiorum*, was shown to internalize sEVs of *Arabidopsis thaliana* containing small RNA (sRNA) to target fungal genes involved in virulence and secretion (Cai *et al*. 2018). It is not understood how uptake of plant EVs by fungal spores occurs (Regente *et al*. 2017).

Uptake of EVs by plant cells remains even more mysterious. However, uptake of garlic-derived nanovesicles by liver cells was shown to involve the interaction between the transmembrane glycoprotein heterodimer CD98 and a mannose-binding lectin (Song *et al*. 2020). This finding suggests that similar interactions between glycoproteins and lectins could play a role in EV uptake by plant and fungal cells.

### Walking through walls: EV release and uptake in bacteria, fungi and plants

Unlike animal cells, bacteria, fungi and plants contain cell walls that may interfere with secretion, delivery and uptake of EVs. Despite the conceived physical restrictions of cell walls, it is now appreciated that all organisms with cell walls are able to produce and, in the case of fungi, take up EVs. Although the model organism *S. cerevisiae* has extensively been used to study secretion, fungal EVs were first observed experimentally in the opportunistic pathogen *C. neoformans* (Takeo *et al*. 1973). These early freeze-dried EM studies, replicated in *C. albicans* (Anderson, Mihalik and Soll 1990) and *S. cerevisiae* (Osumi 1998), depict various vesicular structures penetrating and emerging from the cell wall. To date, EVs have been identified in many clinically relevant genera, including Histoplasma, Paracoccidioides, Sporothrix, Candida, Malassezia, Aspergillus and Fusarium (Bielska and May 2019).

Much attention has been given to the problem of how vesicles traverse thick cell walls, such as those found in fungi, mycobacteria, Gram-positive bacteria and plants. With regards to fungi, speculations have ranged from turgor pressure forcing vesicles through the wall, to enzymatic cell-wall modification, as well as transit through channels, allowing vesicles to 'walk through the wall' (Brown *et al*. 2015). While some of these conjectures await corroboration, a number of experiments have shown a way through. First, degradative and remodelling enzymes have indeed been recurrently found in a range of fungal EVs (Albuquerque *et al*. 2008; Zhao *et al*. 2019; Karkowska-Kuleta *et al*. 2020). Second, cell-wall pore size on the surface of *S. cerevisiae* has been shown to fluctuate between 50 and up to 400 nm when under stress (de Souza Pereira and Geibel 1999), suggesting a gating method. Factors impacting pore size include osmotic changes (Garcia-Rubio *et al*. 2019), oxidative stress (de Souza Pereira and Geibel 1999) and stage in the cell cycle (Gow and Hube 2012). Moreover, it is likely that EVs, themselves morphologically dynamic, pass through pores much smaller than their spherically idealized diameter (Brown *et al*. 2015). In *C. neoformans* melanization was shown to decrease porosity, causing vesicles to accumulate between the plasma membrane and cell wall (Jacobson and Ikeda 2005).

While much of this research has been done on budding yeast and *C. neoformans*, the implications are far-reaching in that the latter fungus has a filamentous stage and cell walls of all fungi have similar composition, consisting of mannoproteins, β-glucans and chitin (Brown *et al*. 2015). Although cell wall composition differs in oomycetes in that they do not produce chitin, their pore sizes of cell walls are equally tiny being impermeable to molecules with diameters in excess of 2–3 nm (Money 1990). Estimated pore sizes of plant cell walls are 5–7 nm in diameter based on the permeability of globular proteins of 36–67 kDa (Fry 2017). However, it has been appreciated that plant cell walls are dynamic (Greve and Labavitch 1991; Rose *et al*. 1998) and plant cells have been shown to secrete much larger molecules (Fry 2017). It is well possible that the dynamic cell wall through interaction with EVs facilitates their passage through the assistance of cell wall modifying enzymes as outlined in this treatise.

### Fungal EVs as virulence factors

Since mutants with impaired EV secretion exhibit reduced fitness, and application of additional EVs increases infectivity, there is strong evidence associating fungal EVs with virulence (Panepinto *et al*. 2009; Huang *et al*. 2012; Wolf *et al*. 2015). A diverse range of macromolecules are featured in fungal EVs, with roles in virulence, signalling, scaffolding and metabolism, including proteins, lipids, nucleic acids, glycans, pigments and sterols (Kitajma 2000; Bleackley *et al*. 2019). Moreover, the EV profile can vary according to environmental conditions, such as the relative availability of nutrients (Cleare *et al*. 2020), host immune response (Vargas *et al*. 2015) and potentially quorum sensing (Padder, Prasad and Shah 2018). Indeed, EVs derived from *C. albicans* biofilm comprise a single population with ESCRT proteins implicated in their biogenesis, whereas planktonic EVs are more polydisperse in size with a bimodal distribution, indicating distinct subpopulations (Zarnowski *et al*. 2018). Such data resembles recent work in model bacterial organism *Pseudomonas aeruginosa*, showing quorum-dependent biofilm EVs to differ in profile from planktonic EVs (Cooke *et al*. 2019), thus highlighting the evolutionary conserved relationship between EVs and biofilm.

Fungal EVs may be internalized by host immune cells via endocytic pathways (Fig. 3D). Fungal EVs provoke strong animal immune responses *in vitro* and *in vivo*, offering the potential for mycosis vaccines (Freitas *et al*. 2019). Common EV-associated immunogens include cell-surface PAMPs such as membrane-bound glycan moieties. In particular those of *Paracoccidioides brasiliensis* and *P. lutzii* have been characterized as being recognized by C-type lectin receptors (CLR) found on the surface of macrophages and dendritic cells (Peres da Silva *et al*. 2015a). Similarly, lipid components of the cell wall present in EVs, such as glucosylceramide, have been shown to bind IgG2a monoclonal antibodies (Toledo *et al*. 2001).

As with much early immunological work, the story of inflammatory mediators remains somewhat convoluted, however, some consistency has been shown across fungal species with EVs isolated from *Aspergillus flavus* (Brauer *et al*. 2020),*Trichophyton interdigitale* (Bitencourt *et al*. 2018) and *Paracoccidioides brasiliensis* (da Silva *et al*. 2016) all inducing macrophage polarization to M1 *in vitro*. Acute-phase pro-inflammatory tumour necrosis factor α (TNF-α) also appears to be broadly released from professional antigen presenting cells when in the presence of EVs from *C. albicans*, *Malassezia* spp., *T*. *interdigitale* and *Sporothrix brasiliensis* (Campos *et al*. 2015; Bielska and May 2019). Much of the work in this area (Freitas *et al*. 2019) suggests a nuanced interplay between immunostimulatory and immunosuppressive effects, with elevated nitrous oxide (NO) and the cytokines IL4, IL10, IL12, TGF-β, IL6, IL12 and IFNγ featuring frequently.

For example, there has been ongoing debate as to whether EVs act deleteriously on the host immune system or otherwise provide beneficial challenge. In the well-studied case of opportunistic fungal pathogen *C. neoformans*, EVs harbour the capsular antigen glucuronoxylomannan (GXM), which can suppress monocytes, neutrophils and T lymphocytes (Monari, Bistoni and Vecchiarelli 2006) and has been shown to confer cytotoxic effect directly to macrophages via the Fas/FasL pathway (Villena *et al*. 2008). These EVs induce macrophages to produce anti-inflammatory TGF-β and IL-10 *in vitro*, while conversely stimulating via TNF-α (Oliveira *et al*. 2010a). In the search for fungal EV-based vaccines, it has been shown that *C. neoformans* mutants lacking wild-type GXM fail to generate a protective immune response in a murine vaccination model, whereas GXM-containing EVs stimulated resistance to infection in a *Galleria mellonella* model (Colombo *et al*. 2019). Based on these observations, it was suggested the host-protective effects of EVs may outweigh pathological effects (Freitas *et al*. 2019). However, the evidence is thus far insufficient to guide clinical practice. Similarly, *Malassezia sympodialis* releases allergenic EVs, which induce high levels of both the pro-inflammatory TNF-α and the anti-inflammatory IL-4 and are consequently associated with a possible dual immunoregulatory function in atopic eczema (Gehrmann *et al*. 2011).

In a vivid example of cross-kingdom communication, evidence indicates that *C. neoformans* EVs enhance brain infection by facilitating the crossing of the blood–brain barrier and modulate antimicrobial action of the host by inducing cytokines (Huang *et al*. 2012). Bolstering the view that EVs can act as immunological effectors at great distance, recent work shows that EVs isolated from virulent strains of *C. gattii* are readily taken up by macrophages, stimulating the rapid growth of less-virulent, intracellular, non-outbreak fungal cells that would otherwise be degraded (Bielska *et al*. 2018).

### Be quiet! EVs and cross-kingdom RNA silencing

For reasons outlined in the previous section, EVs are uniquely positioned as vectors for cross-kingdom RNA interference (RNAi) dissemination by providing protection from enzymatic degradation and opportunities for cell targeting (Fire *et al*. 1998; Cheng *et al*. 2014). Phrased inversely, RNA may not get very far, particularly outside host cells, without encapsulation by EVs. This view has been challenged, as extracellular RNAs are also stabilized by RNA-binding proteins (RBPs), such as nucleophosmin 1 (NPM1) and argonaute 2 (AGO2) (Wang *et al*. 2010b; Zhao *et al*. 2019). Others point out that such RBPs are often associated with loading of RNA into EVs, and so may be supportive, rather than alternative (Leidal *et al*. 2020; Xu *et al*. 2020).

RNAi and EV biogenesis are suggested to be linked, based on work in animals showing that depleting ESCRT proteins to block MVB formation results in impaired miRNA silencing and loss of the cytoplasmic foci known as P-bodies, where many of the RNA-induced silencing complex (RISC) proteins necessary for silencing are localized (Lee *et al*. 2009). Microarray and bioinformatic analysis of RNA extracted from primary T lymphoblast sEVs revealed a common sequence motif, named the EXOmotif, found only in vesicular sRNA. Importantly, mutagenesis of this motif inhibited packaging into sEVs and introduction of this motif into non-consensus miRNAs stimulated sEV release (Villarroya-Beltri *et al*. 2013). However, miRNAs without an EXO motif are also found in EVs, so further proteins have been sought, with the Y-box protein 1 (YBX1) being identified in tetraspanin (CD63)-positive sEVs and subsequently implicated in EXO-independent secretion (Shurtleff *et al*. 2016), although others (Jeppesen *et al*. 2019) were unable to reproduce this finding. While genetic screenings have largely been overlooked as interrogative tools for elucidating RNA packaging in EVs, an innovative CRISPR-Cas9 miRNA barcoding strategy was applied to corroborate established EV-supporting genes, such as Rab27a and sphingomyelinase, and identify novel contributors, specifically the role of the Wnt signalling pathway (Lu *et al*. 2018). How translatable this work is to other kingdoms of life remains to be seen.

### Role of EVs in the virulence of phytopathogenic fungi

Little is known about the role of phytopathogenic EVs in fungal virulence. EVs isolated from in the axenically grown wheat pathogen *Zymoseptoria tritici* contained relatively few carbohydrate-active hydrolytic enzymes, proteases and effectors relative to conventionally secreted proteins (Hill and Solomon 2020). Nevertheless, the cotton pathogen *Fusarium oxysporum* f. sp. *vasinfectum* releases EVs in liquid cultures that contain a purple pigment and trigger a phytotoxic response when infiltrated into leaves (Bleackley *et al*. 2019). It was also mentioned in this article that *M. oryzae* delivers effectors via the noncanonical effector secretion pathway to the BICs (Giraldo *et al*. 2013).

**Figure 4.**
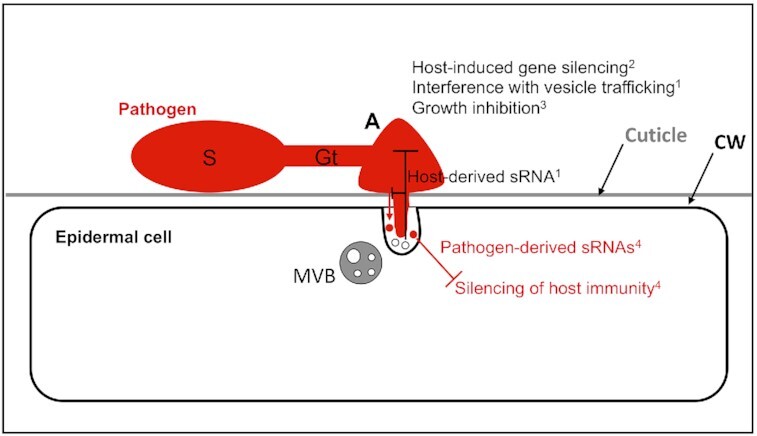
Cellular communication between an invading filamentous pathogen and an epidermal cell of a host plant; the fungal spore (S) has attached to the plant surface that is covered and impregnated by a cuticle in aerial plant parts. Host surface and chemical cues facilitate the formation of a germ tube (Gt) and appressorium (A), which generates pressure and hydrolytic enzymes to break down the plant cell wall (CW) and penetrate the host epidermal cell with a penetration peg. Part of the penetration resistance pathway of host plants is the generation of MVBs and release of exosomes (putative sEVs) at the site of pathogen invasion. Among other molecules, these putative sEVs contain sRNAs that can target microbial components of vesicle trafficking ^1^(Cai *et al*. 2018). This form of plant immunity can inhibit the production of pathogen-derived sRNAs that may target and silence host immunity genes ^4^(Weiberg *et al*. 2013). Exosomes, reminiscent of sEVs, from plants can also inhibit fungal growth ^3^(Regente *et al*. 2017) and stall further ingress. Plant EVs may also contribute to host-induced gene silencing ^2^(Koch and Kogel 2014). Cellular structures are not drawn to scale.

During plant–pathogen interactions, sRNAs are exchanged to execute cross-kingdom/organism RNA interference (Fig. 4). The fungal pathogen *Botrytis cinerea*, for instance, delivers sRNAs to silence corresponding host target genes involved in immunity (Weiberg *et al*. 2013). Conversely, the host plant *A. thaliana* generates sRNAs that target fungal genes involved in vesicle trafficking to reduce pathogen virulence (Cai *et al*. 2018). These plant-derived sRNAs are found in a subpopulation of EVs, i.e. tetraspanin-containing sEVs (Table 1). These putative sEVs contain the RNA-binding proteins argonaute 1 (AGO1), RNA helicases (RH) and annexins (ANN) to facilitate sRNA loading and/or stabilization (He *et al*. 2021). Whereas AGO1, RH11 and RH37 selectively bind to EV-enriched sRNAs, ANN1 and ANN2 bind sRNAs nonspecifically. The *rh11 rh37* and *ann1 ann2* double mutants of *A. thaliana* were hypersusceptible to *B. cinerea*. Plant EVs were recently shown to contain 'tinyRNAs' 10–17 nucleotides (nt) in length, and the presence of 21–24 nt long sRNA in EVs was challenged (Cai *et al*. 2018; Baldrich *et al*. 2019). Some miRNAs and secondary siRNAs are enriched in the apoplastic fluid but not in EVs, suggesting EV-independent sRNAs secretion pathways (Baldrich *et al*. 2019). EVs accumulating at the haustorial interface may well contribute to fungal EV-mediated RNAi (Bozkurt and Kamoun 2020).

### Use of RNAi for plant biotechnology

Host-induced gene silencing (HIGS) has been developed as a novel alternative crop protection strategy against pathogens and pests (Koch and Kogel 2014). Recently, EVs from transgenic *A. thaliana* expressing noncoding dsRNA have been shown to concomitantly silence two fungal cytochrome P450 genes and inhibit growth in *Fusarium graminearum*, both *in vitro* and *in planta*; notably, ESCRT-III mutants were further shown to be HIGS impaired (Koch *et al*. 2020).

### Vesicle trafficking during host–pathogen interactions

When fungal spores first land on plant surfaces, they produce adhesives to avoid displacement as a result of the physical environment or microbial competition (Tucker and Talbot 2001). In the case of rust fungi, thigmotropism is sufficient in the absence of chemical sensing for germ tube and appressorium formation (Hoch *et al*. 1987). Other fungal pathogens, like *Colletotrichum gloeosporioides*, depend on host chemicals, e.g. wax components, for appressorium formation (Podila, Rogers and Kolattukudy 1993). However, little is known about the exchange of information via plant and fungal secretion pathways until the stage of pathogen penetration of host cells.

The powdery mildew fungus *Blumeria graminis* f. sp. *hordei* (*Bgh*) produces a primary germ tube after attachment to the barley leaf surface that penetrates the cuticle. This penetration event triggers H_2_O_2_ accumulation and formation of a papilla containing host cell wall appositions (Huckelhoven and Kogel 2003). However, this early event does not determine the outcome of this host–pathogen interaction. Instead, it is the host response to a second appressorial germ tube that determines whether the interaction will be compatible or incompatible (Collins *et al*. 2003).

### Resistance of plants against penetration by pathogens

Resistance against powdery mildew fungi is associated with an apoplastic immune response of epidermal cells, which generates papillae underneath fungal contact sites through appositions between the cell wall and the plasma membrane (An *et al*. 2006b). These papillae contain callose, a ß-1,3-glucan plant cell wall polymer, generated by the enzyme PMR4/GSL5 (Jacobs *et al*. 2003; Nishimura *et al*. 2003). Callose deposition was monitored in a mutant screen for nonhost resistance of *A. thaliana* against the nonadapted pathogen *Bgh*, resulting in identification of the syntaxin or t-SNARE *SYP121*/*PEN1*; its ortholog *Required for* mlo-*specified resistance2* (*ROR2*) contributes to basal penetration resistance of barley against the same pathogen (Collins *et al*. 2003). Plasma membrane-localized syntaxin ROR2 and its interacting partner HvSNAP34 are thought to facilitate exocytosis or homotypic fusion of vesicles (Pickett and Edwardson 2006). Vesicle fusion is driven by complex formation of t-SNAREs and v-SNAREs through vesicle-associated membrane proteins (VAMPs). PEN1 and SNAP33 form a SNARE complex with VAMP721 and VAMP722 (Kwon *et al*. 2008). GFP-tagged VAMP722 accumulates in intracellular compartments of varying sizes, reminiscent of mammalian compound exocytosis with vesicles fusion prior to their secretion (Pickett and Edwardson 2006). Callose deposition was found to occur independently of coincident entrapment of PEN1 and its interacting partner SNAP33 in the papillary matrix (Meyer *et al*. 2009).

MVBs accumulate beneath the appressorial penetration peg to release sEVs containing H_2_O_2_, Ca^2+^, peroxidase PRX7 and phenolic compounds into papillae (An *et al*. 2006a; An, van Bel and Hückelhoven 2007); the MVB marker ARA6 accumulates near fungal attack sites (Nielsen *et al*. 2012). Callose, however, accumulates in cell wall appositions and not in MVBs, consistent with localization of callose synthase to the plasma membrane (An *et al*. 2006a). Moreover, the ß-glucosyl hydrolase PEN2 and the ABC transporter PEN3 act in a pathway to synthesize and transport antimicrobial compounds across the plasma membrane into extracellular encasements that surround and delimit haustoria of powdery mildew fungi (Lipka *et al*. 2005; Stein *et al*. 2006). MVBs also accumulated in cells neighbouring infected cells undergoing hypersensitive cell death to constrict or block plasmodesmata with cell wall appositions (An *et al*. 2006b); this reaction serves to stop communication between neighbouring cells and contain cellular damage. During compatible interactions, the penetration peg differentiates into a haustorium, a specialized feeding structure surrounded by an extrahaustorial plant membrane that features membrane evaginations (An *et al*. 2006b) reminiscent of mEVs (Fig. 2; Table 1). MVBs and other vesicles also accumulate in the vacuole (An *et al*. 2006b), suggesting the occurrence of autophagy (Table 1) (An *et al*. 2006a).

MVBs and post-Golgi vesicles are essential for plant defence against host cell-penetrating fungal pathogens. A putative component of the vesicle tethering complexes (*HvEXO70F-like*), a subunit of the conserved oligomeric Golgi (COG) complex (*HvCOG3*) and a MVB/trans-Golgi network-localized ADP ribosylation factor (ARF) GTPase (*HvARFA1b/c*) are involved in resistance of barley against *Bgh* (Table 1) (Ostertag *et al*. 2013). Moreover, accumulation of ARFA1b/1c-positive MVBs near fungal penetration sites is necessary for PEN1 accumulation in sEVs destined to reach papillae and for callose deposition (An *et al*. 2006a; Meyer *et al*. 2009; Bohlenius *et al*. 2010). Likewise, callose and GFP-PEN1 deposition in the papillary matrix is dependent on the large ARF-GTP exchange factor (ARF-GEF) GNOM (Nielsen *et al*. 2012). It was suggested that GNOM-mediated trafficking recycles preexisting plasma membrane material, e.g. PEN1, to papillae for penetration resistance (Meyer *et al*. 2009).

### Trade-off between penetration resistance against powdery mildews and defence against other pathogens

Mutations in the *mildew resistance locus o* (*Mlo*) of barley confer durable resistance against powdery mildew pathogens (Kusch and Panstruga 2017) but increase susceptibility to *Magnaporthe grisea*. However, this increased susceptibility was independent of the *ROR1* gene in contrast to the requirement of *ROR* genes for *mlo* resistance against *Bgh* (Jarosch, Kogel and Schaffrath 1999). The *mlo5* mutant is also more sensitive to *Bipolaris sorokiniana* toxin, a response dependent on *ROR1* and correlated with an overaccumulation of H_2_O_2_ (Kumar 2001). Barley *mlo* mutants are also more susceptible to the head blight pathogen *Fusarium graminearum* (Jansen *et al*. 2005). In field trials and seedling assays, *mlo* alleles increased the severity of Ramularia leaf spot disease caused by the pathogen *Ramularia collo-cygni* (McGrann *et al*. 2014). Disease symptoms were reduced in *mlo5 ror1* and *mlo5 ror2* double mutants but fungal biomass remained as high as in *mlo* single mutants, indicating that *ROR* genes regulate the transition from endophytic to necrotrophic colonization. Conversely, *mlo5* mutants did not affect *Fusarium* spp. and *R. collo-cygni* colonization compared with corresponding wild-type cultivars in independent field trials (Hofer *et al*. 2015), suggesting that environmental conditions may have an influence on this trade-off. Collectively, these findings suggest that host EV release may mediate resistance or susceptibility depending on particular pathogen penetration and colonization strategies.

Vesicle secretion may have different consequences depending on the pathogen that is being encountered by a specific host. In response to powdery mildews, EVs may protect the host against pathogen ingress. Other pathogens, however, may be stimulated to infect and colonize when receiving information from the host in form of secreted vesicles. The cargo of EVs may differ depending on whether a deterring or stimulating activity exists, but this has not yet been investigated. Environmental factors may influence the crosstalk that exists between hosts and pathogens under different field settings. Much needs to be explored regarding the details of vesicle trafficking affecting different outcomes.

### Evidence for a role of the exocyst in innate plant immunity

Other *Exo70* genes than the ones mentioned earlier are involved in defence against microbial pathogens. Exo70B2 was identified as a target of the plant U-box-type ubiquitin ligase 22 (PUB22), which together with PUB23 and PUB24 down-regulates pathogen-associated molecular pattern (PAMP)-triggered immunity (Trujillo *et al*. 2008). Accordingly, *exo70B2* mutants were hyper-susceptible to the virulent bacterial pathogen *Pseudomonas syringae* pv. *tomato* (*Pst*) DC3000 and obligate biotrophic downy mildew oomycete pathogen *Hyaloperonospora arabidopsidis* (Stegmann *et al*. 2012). *Exo70B1*, on the other hand, is involved in pathogen-specific immune responses; *exo70B1* mutants were lesion mimics with increased susceptibility to *Pst* DC3000 but elevated resistance against *H. arabidopsidis* (Stegmann *et al*. 2013). These different phenotypes may be reconciled because *Exo70B1* has been shown to be involved in autophagy-related trafficking to the vacuole (Kulich *et al*. 2013). Within this context, the contribution of autophagy to the negative regulation of resistance (R) protein receptors that interact with corresponding pathogen effectors (Yoshimoto *et al*. 2009; Pecenkova *et al*. 2016) is of significance. The role of *Exo70H1* in pathogen defence has also be tested but with less conclusive results, partially because of the redundancy with *Exo70H2* (Pecenkova *et al*. 2011). Detailed analysis of *Exo70* gene family members in vesicle trafficking during pathogenesis is still needed (Vukasinovic and Zarsky 2016), even though some probably contribute to the tethering of MVBs to the plasma membrane for release of sEVs. As mentioned, plants produce EXPO-derived EVs (Wang *et al*. 2010a), but their generation during immune responses still needs to be investigated.

### Vesicle secretion in phytopathogens during host penetration and colonization

Appressoria and haustoria of *Bgh* generate MVBs (Table 1) (An *et al*. 2006b). The 'punchless' mutant of *M. grisea* is able to generate appressoria but unable to penetrate rice leaves (Clergeot *et al*. 2001). The inactivated *PLS1* gene encodes a tetraspanin, which is a known marker for sEVs (Inal *et al*. 2013b). It would be desirable to determine the production of sEVs in the wild type and the 'punchless' mutant of *M. grisea* (Table 1).

Uncoated and coated vesicles were observed in epidermal cells invaded by infection vesicles of *Colletotrichum lindemuthianum* (Mengden and Deising 1993), suggesting membrane recycling and vesicle secretion at the initial cellular contacts between the invading pathogen and its bean (*Phaseolus vulgaris*) host. Later stages of colonization by *M. oryzae* involve vesicle trafficking. Upon penetration of epidermal cells, the fungus forms IH that secrete apoplastic effectors like Bas4 via the conventional secretion pathway. IH form BICs in a newly infected rice cells. BICs accumulate cytoplasmic effectors like Pwl2 that are destined for translocation into host cells via an unconventional secretion pathway; this type of secretion is dependent on exocyst and t-SNARE complexes (Giraldo *et al*. 2013). Autophagy was recently shown to maintain the biotrophic phase of *M. oryzae*; *Δimp1* mutants were impaired in maintaining BICs as evidenced by loss of Pwl2 expression and cytoplasmic accumulation of the apoplastic effector Bas4 (Table 1) (Sun *et al*. 2018).

### Plant–pathogen communication with EVs

The abundance of EVs collected from apoplastic fluids of *A. thaliana* leaves increased after infection with *Pst* DC3000 or treatment with salicylic acid (Rutter and Innes 2017). The protein composition of these EVs suggests that they probably represent sEVs that are enriched for PEN1.

Apoplastic fluids from bean leaves induce excision of a 106 kb genomic island from the chromosome of *P. syringae* pv. *phaseolicola* containing the effector gene *avrPphB* (Godfrey *et al*. 2011). The generated circular episome suppresses expression of *avrPphB*, thus preventing its recognition by the *P. vulgaris* receptor encoded by the corresponding *R3* gene (Pitman *et al*. 2005). It remains to be determined which apoplastic molecules or EVs may be involved in bacterial recognition and generation of this stealth episome.

Viral infection and spread in plants differs from animals (Gray and Banerjee 1999). Most plant viruses are insect-borne. For instance, phloem-feeding insects will deliver viruses into host cells, from which systemic spread occurs through plasmodesmata from cell to cell via the symplastic route. This can occur via viral ribonucleoprotein complexes or entire virions (Niehl and Heinlein 2011). An exception to this rule was recently published, demonstrating that turnip mosaic virus can be secreted via sEVs in form of double-stranded RNA or viral replication complexes (Movahed *et al*. 2019). The onset of viral secretion occurs at the ER with assembly of replication complexes into vesicles that bypass the Golgi apparatus to reach MVBs (Wang *et al*. 2010a). Viral delivery into the apoplast can explain the presence of replication vesicles in xylem vessels that spread viral infection even after girdling of the stem (Wan *et al*. 2015). sEVs are also essential for release of rice dwarf virus from insect vector cells through fusion of MVB and the plasma membrane (Wei, Hibino and Omura 2009).

### Animal EVs during attack

Unlike plants, the immune system of animals relies on phagocytosis (Stotz *et al*. 2003). This may be one of the reasons why EVs do not function as defence compartments in animals as they do in plants during the abovementioned penetration resistance. Despite little comparison of fungal pathogenesis of plants with that of animals in the scientific literature, there are obvious differences.

First, penetration or uptake by fungal pathogens of host plant and animal cells reflects the differing challenges posed. Whilst penetration of plant cells may require mechanical pressure, proteolytic degradation or enzymatic degradation of cell walls as well as entry through stomata, animal pathogens such as *Histoplasma capsulatum* are taken up by receptor-mediated endocytosis (Table 1) (Garfoot and Rappleye 2016).

Second, in animal (vertebrate) species, the immune system stimulates inflammation, attracting cells to control infection. As plants cannot recruit cellular immune effectors, an invading hypha interacts with a solitary plant cell. In the case of biotrophic pathogens, such infection thus triggers programmed cell death of the challenged plant cell, in an attempt to stave off infection. With no cell-mediated immunity, plant EVs with antifungal properties, released into the apoplastic space, take up this role (Regente *et al*. 2017; Cai *et al*. 2018). In animals, there are many EV-mediated interactions between host and pathogen (Inal, Ansa-Addo and Lange 2013a). Natural killer (NK) cells kill virally infected cells by inducing apoptosis; they also kill fungi directly (Table 1) (Schmidt, Tramsen and Lehrnbecher 2017) as well as cancer cells. Released perforins allow granzymes to enter the infected cell inducing apoptosis; this also occurs upon interaction of death receptor ligands with death receptors such as Fas. This killing mechanism (although research has focused on cancer cells) can also be mediated by EVs released from the NK cell (Di Pace *et al*. 2020). EVs derived from another innate immune cell, the cytotoxic CD8^+^ T-cell, may also mediate cell death as well as activate other immune cells (Table 1) (Del Vecchio *et al*. 2021).

## CONCLUDING REMARKS

**Figure 5.**
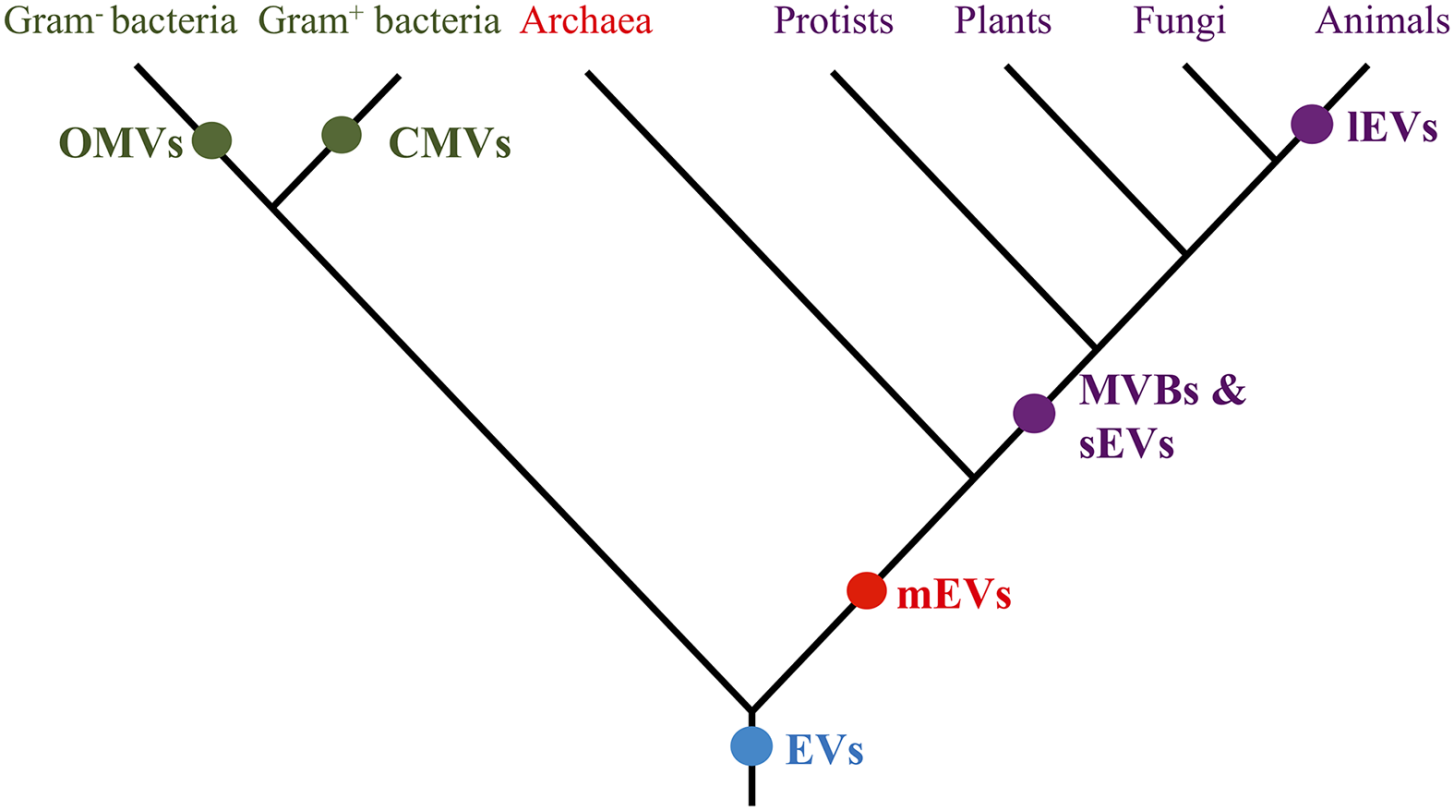
Hypothetical evolution of EVs in all forms of life; the three domains of life (bacteria, archaea and eukaryotes) produce EVs; this suggests that the LUCA already produced EVs. Gram-negative bacteria produce OMVs, specialized EVs that contain components of the periplasmic space rather than cytoplasmic molecules, and outer–inner membrane vesicles with cytoplasmic content. Gram-positive bacteria produce cytoplasmic membrane vesicles (CMVs). Archaea and eukaryotes share an endosomal sorting complex required for transport (ESCRT) to produce medium EVs (mEVs or microvesicles) also referred to as membrane exfoliated vesicles. Small EVs (sEVs or exosomes), derived from MVBs, are specific to eukaryotes. Apoptosis sensu stricto only occurs in animals, generating large EVs (lEVs or apoptotic bodies). mEVs are not well documented in plants and fungi perhaps due their rigid cell walls. Unlike animal viruses, most plant viruses spread through plasmodesmata and do not bud from the host plasma membrane. This model is supported by Gill, Catchpole and Forterre (2019).

EVs contribute to cellular functions of all living organisms (Fig. 5). It is therefore hypothesized that EVs are conserved and have been produced by the earliest living cells. This thermodynamically underpinned biophysical process is thought to have evolved over time to incorporate complex regulatory control to prevent random fusion events and allow selective packaging and enrichment of potent effectors in response to external stimuli. This in turn allows for remote dissemination of virulence factors.

Prokaryotic EVs are not the topic of this article, but production of OMVs by Gram-negative species and the more recently identified cytoplasmic membrane vesicles (CMVs) of Gram-positives suggests that the last common ancestor of all three domains of life produced EVs (Gill, Catchpole and Forterre 2019; Toyofuku, Nomura and Eberl 2019). In addition to the biogenesis of OMVs through blebbing of the outer membrane, Gram-negative bacteria generate outer-inner MVs (OIMVs) and explosive OMVs (EOMVs) after bacteriophage-induced explosive lysis (Toyofuku, Nomura and Eberl 2019). These OIMVs and EOMVs contain cytoplasmic macromolecules. Similarly, CMVs of Gram-negative bacteria also contain cytoplasmic components because they do not contain an outer membrane and periplasmic space. Prokaryotic vesiculation is shown to be involved in generalized secretion, virulence and membrane remodelling, as well as specific envelope stress responses, biofilm development, horizontal gene transfer, phage receptor transfer and extracellular scaffolding (Kulp and Kuehn 2010; Manning and Kuehn 2011; Guerrero-Mandujano *et al*. 2017).

Some archaeal genomes encode ESCRT III proteins and/or Vps4 ATPases to produce mEVs (Ellen *et al*. 2009). Based on this primordial ESCRT complex (Spang *et al*. 2015), it is parsimonious to propose that mEVs are evolutionarily old and plasma membrane blebbing is a more ancient mechanism of EV production. Of note, bacterial MV production is also based on membrane blebbing. In contrast, MVBs and sEVs are suggested to be a more recent evolutionary invention of eukaryotes because the machinery required for generating MVBs and sEVs is more complex, requiring ESCRT I-III, and not yet present in archaea. In addition, tetraspanins, which are found in sEVs, are limited to all eukaryotic cells and not found in archaea (Huang *et al*. 2005). Membrane blebbing, biogenesis of lEVs, is a hallmark of apoptosis found only in animals (Kutscher and Shaham 2017). Although programmed cell death does occur in other eukaryotes, all morphological and molecular hallmarks like caspases are only present in animals (Koonin and Aravind 2002). It is therefore suggested that lEVs recently developed in the animal kingdom (Fig. 5). Of note, lEVs can contain entire organelles (Lieberthal and Levine 1996); this does not happen in mEVs.

In eukaryotes, the array of uses and manner in which EVs are produced and processed has expanded further to include complex immunomodulatory interactions as well as widespread RNA silencing via miRNAs, siRNAs, amongst others. This appears to be conserved across all four kingdoms. Fungal EVs have been shown to be immune-modulatory in animals. In animal cells, EVs are implicated in modulation of both the pre-metastatic niche and cancer microenvironment. Fungal and plant cell walls may restrict movement of mEVs and lEVs. The pathways leading to their biogenesis may therefore be deemphasized when cell walls with small apparent pore sizes exist. However, when cell wall degradation occurs, production of mEVs may become important (An *et al*. 2006b; Rizzo *et al*. 2020).

Table 1 adds to a final summary of published findings and interpretations related to plant and microbial interactions. Vesicular secretion contributes to compatible interactions and pollen hydration as the first step during flower fertilization. In contrast, incompatible interactions between host plants and powdery mildew fungi are dependent on the secretion of sEVs for penetration resistance. Microscopic and genetic evidence suggest that autophagy occurs during plant–pathogen interactions. Fungal autophagy is specifically needed for prolonged compatible interactions with *M. oryzae*. At the same time, fungal secretion appears to be essential for fungal infection and colonization of the host. Vesicle trafficking may therefore be complex and serve dual needs. The same may be happening in host plants to moderate the immune response while combating pathogens. Trade-offs exist during defence against different pathogens and there may be an environmental influence on these trade-offs. Secretion of EVs by host plants may therefore not always be directed for defence but also facilitate compatible interactions. Plant developmental and immune reactions may therefore have similarities after all.

## ACKNOWLEDGEMENTS

Ralph Hückelhoven kindly provided an EM image and a cartoon to generate one of the figures for this article. Thanks are expressed to Nick Talbot (Sainsbury Laboratory) and Dawn Arnold (UWE Bristol) for inspirational discussions at BSPP meeting in 2019. John Labavitch (UC Davis) is posthumously acknowledged for his expertise and insights into the dynamics of plant cell walls and their variable pore sizes.
